# A Systematic Review of Tools to Assess Coeliac Disease-Related Knowledge

**DOI:** 10.3390/jcm13144053

**Published:** 2024-07-11

**Authors:** Sophie Hall, Kristin Kenrick, Andrew S. Day, Angharad Vernon-Roberts

**Affiliations:** 1 Department of Paediatrics, University of Otago Christchurch, 4 Oxford Terrace, Christchurch 8011, New Zealand; sophie.hall@postgrad.otago.ac.nz (S.H.);; 2 Department of Paediatrics, Christchurch Hospital, 2 Riccarton Ave, Christchurch 8011, New Zealand; 3 Department of General Practice and Rural Health, Dunedin School of Medicine, Rm 124, 55 Hanover Street, Dunedin 9016, New Zealand

**Keywords:** coeliac disease, knowledge assessment, questionnaire, survey, validity, reliability

## Abstract

**Background**: Coeliac disease (CD) is an immune-mediated disorder, with dietary exclusion of gluten the only current treatment. A good knowledge of CD and gluten-free diet (GFD) is essential for those with CD to support effective self-management. Knowledge assessment with a validated tool helps evaluate understanding and knowledge gaps to better tailor educational resources. This study’s aim was to perform a systematic review to identify validated CD knowledge assessment tools. **Methods**: PRISMA guidelines were followed, and searches were carried out in five literature databases. Papers were reviewed for tool development and testing process and assessed against pre-defined criteria for feasibility, validity, and reliability. **Results**: Twenty-five papers were included in the final analysis. Studies were from 16 countries, with a range of target populations, study designs, and development processes. Eleven reported pilot testing, and five assessed readability. Content validity was assessed in ten papers and formal content validity testing in one. Many tools contained items affecting generalisability outside the region developed. **Conclusions**: For a CD knowledge assessment tool to be suitable for use, it needs to be well designed, tested, and generalisable. No papers identified satisfied all requirements, thus highlighting a need to develop an appropriate tool.

## 1. Introduction

Coeliac disease (CD) is an immune-mediated enteropathy that manifests in genetically susceptible individuals as a result of exposure to gluten [1]. It is thought to affect around 1% of the general population worldwide, with increased prevalence seen in certain populations and families [2,3]. These populations include those with concomitant autoimmune disorders, Down syndrome, and Turner syndrome, as well as those with first- and second-degree relatives with CD [2,4,5,6,7].

To date, the only proven treatment for CD involves the strict, lifelong exclusion of sources of gluten from the diet [1]. This requires avoidance of the grains wheat, barley, and rye (and their relatives), a staple of the diet in many countries [1]. Many countries also recommend that oats are also excluded due to the high likelihood of cross-contamination, as well as a small subset of those with CD having an increased sensitivity to the similar proteins in some varieties of oats [8,9]. Strict avoidance of gluten is essential in order to attenuate risk of disease-related complications, with even small amounts of gluten being harmful to some individuals [10]. This strict avoidance can be challenging due to the risk of cross-contamination with gluten-containing foods, changes to the ingredient lists of commercial products, hidden sources of gluten in the diet, and the often-higher cost of gluten-free alternatives [11]. Strict adherence to a gluten-free diet (GFD) requires continued vigilance, with proficient self-management the key to effective management of CD [12]. For individuals with CD, having a sound knowledge and understanding of their condition and the importance of maintaining a GFD is essential for adherence to the diet [13,14]. It is also important for the families of people with CD to understand about CD in order to support them adhering to a life-long GFD [15].

Knowledge levels of people with CD should be formally assessed in order to identify gaps in understanding and to tailor support and education to address these. This assessment should be carried out using a validated tool that is appropriate for the population to be studied. The design of the knowledge assessment tools themselves should be robust to ensure that results obtained from the tool can be confidently relied upon to develop and modify interventions. While a number of CD knowledge assessment tools have been developed and presented in the literature, their applicability to all with CD, as related to children, adults, and those in different countries, is unclear. The overall objective of this study was to undertake a systematic review to identify the different CD knowledge assessment tools that have been developed internationally. The subsequent aims were to analyse tool characteristics to determine which tools are valid, reliable, and generalisable for use with different populations with CD in the clinical and research setting. Children were also included in the target audience as knowledge of CD and the ability to self-manage is a gradual process that starts in childhood [16]. Hence, this population could benefit from the use of validated knowledge assessment tools in supporting this process.

## 2. Materials and Methods

### 2.1. Process

This systematic review was performed according to the Preferred Reporting Items for Systematic Reviews and Meta-Analyses 2020 (PRISMA) guidelines [17]. The protocol for this systematic review was not registered with a prospective register of systematic reviews.

### 2.2. Eligibility Criteria

To be included in the review, papers were required to include a written assessment tool to assess knowledge related to coeliac disease. No exclusion criteria were set for population, study type, or publication language in order to ensure identification of all available survey tools.

### 2.3. Information Sources and Search Strategy

A search of the following databases was completed in March 2024: Medline, Embase, Cumulative Index to Nursing and Allied Health Literature (CINAHL), PsychInfo, Scopus and OpenGrey (via DANS). The search strategy included the MeSH and text word terms relating to the condition (coeliac disease), the variable to be measured (knowledge, information), the purpose of the tool (assess, measure), and how the tool may be defined (survey, questionnaire). A full breakdown of search criteria and strategies is included (Appendix A).

### 2.4. Selection and Data Collection Process

The title and abstract of all papers identified under the above search terms were then collated into a database and duplicates removed. This was then independently reviewed by two assessors (SH, AVR) for relevance and inclusion for a full-text review. Any disputed papers were included for full-text review. The full-text versions of the relevant articles were again reviewed independently by two assessors and categorised for inclusion or exclusion with a clear reason for any exclusions. Disputes regarding inclusion were resolved by discussion between all authors (SH, AVR, ASD).

### 2.5. Missing Data

In order to maximise the completeness of the data included, the authors of all papers that had not published their knowledge assessment tool questions were contacted at least twice to provide this information. If a paper included an incomplete assessment tool, the authors were contacted as above. If the full version was not provided, then the sections published were included in the review. Where papers or tools were not published in English, translations were sought from a translator with university-level qualifications in that language.

### 2.6. Data Items and Effect Measures

The details of the different studies were extracted from included papers and displayed in a comparative table. Characteristics included target cohort, country of origin, and study design. A comparative table was compiled looking at the characteristics of the tools identified and the inclusion of considerations relating to health feasibility, validity, reliability, and generalisability. Validated tools to assess these specific metrics are not available; therefore, we based our assessments using the following parameters.

#### 2.6.1. Feasibility

Health literacy: Careful consideration should be made when selecting the type and number of questions for a tool as length and complexity can influence the quality of the data obtained. Questions should be clear and written in language understandable to the target audience.
Readability: Readability assessment ensures that the questions within the tool are written at a level that the targeted reader can fully comprehend. This prevents over-estimation of knowledge deficits due to user misunderstanding of the question rather than its content. It is suggested that health information, particularly when targeting a broad range of literacy levels, be written at no higher than American 6th- to 7th-grade level [18]. This can be assessed using metrics such as the Flesch–Kincaid readability test, whereby a US 6th–7th-grade level equates to a Flesch–Kincaid score of 70–90 [19].
Brevity: Although it is not clear how long an assessment tool should be to optimise data quality, an association has been shown between survey length and response burden, although this must be weighed against development of a questionnaire of sufficient length to answer the intended question [20].
Format: The type of response scales used may affect the amount and type of data that are obtained. For example, Likert scales tend to be used to measure linear agreement with a question, whereas dichotomous Yes/No or multiple choice questions are more commonly used to assess knowledge levels [21].

#### 2.6.2. Validity and Reliability

Following on from feasibility assessment, validation of a tool is essential to ensure that the tool is robust, fit for purpose, and the results obtained are reflective of the function of the tool [22].

Face validity is the more subjective assessment of whether the survey questions accurately measure the intended overall survey question [23]. This can be assessed through expert review and insights based on professional judgement and from participant feedback during pilot testing. However, it is thought to be a somewhat superficial measure of validity, hence not as useful as other forms of validation [24]. Content validity refers to ensuring the inclusion of relevant questions during the design/development phase in order to accurately and comprehensively assess the target metric [25,26]. Content validity during tool development is carried out by literature review and is then structured and undergoes iterative critical analysis by experts [21,26]. Construct validity ensures that the survey is effectively measuring the question [23].

Reliability assessment may be two-fold. The first aspect assesses whether a tool can repeatedly achieve the same results from the same respondents, for example, a test/re-test [23,24]. The second aspect, internal consistency/reliability, relates to how well items in a survey correlate and measure the same general construct, for example, using Cronbach’s alpha [24]. Additionally, inter-rater reliability considers agreement between raters.

#### 2.6.3. Generalisability

The generalisability of survey questions refers to how applicable they are to other/all populations. For example, questions could be specific to regional foods, legislation, or health services and the population that they were administered in and therefore not appropriate for a broader audience.

### 2.7. Synthesis Methods

After tool characteristics were reviewed, all questions included in the tools were extracted and collated into a spreadsheet as outlined above. These questions were reviewed by two assessors and then grouped into broad themes relating to their common knowledge themes, and duplicated questions were combined. From these broad themes, questions were categorised into knowledge domains. Data were then sought on the frequency different questions were asked in the various tools reviewed.

## 3. Results

### 3.1. Study Selection

**Figure 1:**
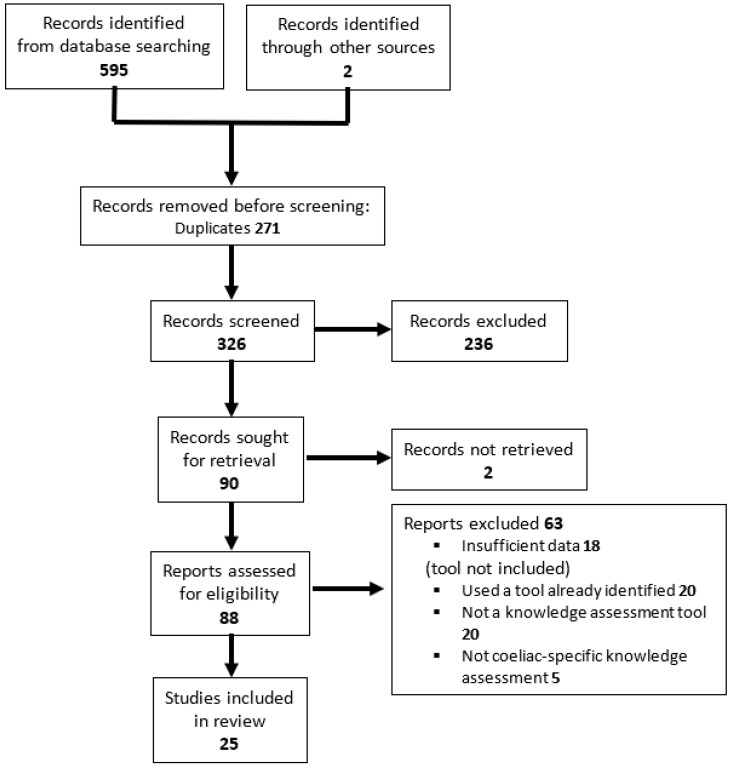
PRISMA 2020 flow diagram of the article selection process.

Initial searches identified 595 publications (Figure 1). Following removal of duplicates (n = 271) and title/abstract review, 90 of these papers met the inclusion criteria for full-text review. Two hundred and thirty-six were excluded at this stage as they did not meet inclusion criteria, (in particular, they did not include a written assessment tool to assess knowledge related to CD).

Of the 90 papers that satisfied criteria for full-text review, a further 65 were subsequently excluded. Twenty papers used a tool that was not looking at the assessment of knowledge in particular, while in eighteen papers a tool could not be accessed after attempting to contact the authors on two or more occasions, or the paper was pending publication. Twenty papers used a tool that had already been identified and the original paper had already been included in the review, and five papers used a tool that was not specific to CD. The remaining 25 papers were included in the systematic review of knowledge assessment tools.

### 3.2. Study Charatcteristics

The 25 studies included in the final analysis were reviewed for their study characteristics (Table 1).

The studies took place in 16 different countries between the years 2003 and 2024. The population sample size and study design varied considerably, from a 20-participant, single-centre study to a 3643-participant, multi-centre study. The majority of the studies were cross-sectional, with the exception of Gutowski et al. [33], Meyer et al. [40], and Vázquez-Polo et al. [47], which included longitudinal data collection, and Vernero et al. [48], who undertook an experimental study. The majority of studies (20 (80%)) looked at knowledge assessments in adults, 2 assessed parental knowledge in parents of children with CD, and 2 studies looked at knowledge in a paediatric population and in adults [30,41,42,49]. Vázquez-Polo et al. [47] planned to assess knowledge of 10–12-year-olds following nutrition education intervention. Knowledge of CD and a GFD was assessed in a range of population groups, with 13 studies among those diagnosed with CD and 7 among healthcare professionals, and 3 studies looked at general public knowledge of CD management and 5 studies among food servers and providers.

### 3.3. Tool Characteristics

#### 3.3.1. Administration

The majority of the tools were self-administered (Table 2), with Garg and Gupta et al. [30] providing a self-completed survey but in an interview setting. Garipe et al. [31] delivered the tool by a trained interviewer. Roma et al. [42] also delivered their tool verbally, adjusting phraseology for age and education level of participants. Eight of the surveys were delivered electronically, with Simpson et al. [44] offering a face-to-face option as well as the electronic survey and one via post [45].

#### 3.3.2. Length

The assessment tools varied in length from 5 to 85 questions (with the 85 questions presented as four question matrices). Six of the knowledge tools sat within larger surveys also looking at other factors such as adherence and attitudes towards the GFD [13,28,30,33,43,44]. Four studies did not use the same tool throughout the study [33,41,44,47]. Riznik et al. [41], Simpson et al. [44], and Tan et al. [45] used slightly different tools for the different target cohorts surveyed. Gutowski et al. [33] administered the same tool for all participants but a different set of questions, in this case a grocery list, at the three timepoints surveyed to assess label-reading comprehension.

#### 3.3.3. Format

A range of different question formats were used in the tools (Table 2). The majority were a dichotomous or Yes/No format with some offering a ‘maybe’ or a ‘don’t know’ option as well. Multiple choice questions were the next most common format with Likert and open-ended questions appearing less frequently. Broadly speaking, questions tended to focus on four main areas: the gluten content of foodstuffs; label reading or identifying gluten in the diet or in medications; CD and its pathophysiology; and assessing the practical daily management of CD and the GFD.

#### 3.3.4. Readability

Readability was mentioned in five of the studies; however, only one provided formal assessment using Flesch–Kincaid readability scores [50]. Howard [36], Silvester et al. [43], and Geiger et al. [32] all included feedback on readability during pretesting and piloting of the tools in peers and neighbours, Canadian Celiac Association members (n = 3), and upper-level dietetic students (n = 16), respectively. Conversely, Roma et al. [42] did not discuss readability assessment during the design phase; however, the questionnaire was delivered by investigators who ‘adjusted phraseology ... as and when necessary’.

#### 3.3.5. Piloting

Eleven studies reported undergoing some form of piloting of their tools prior to use. However, formal piloting was only reported in four studies, with a further six completing some form of piloting with results not reported. Garg and Gupta [30] stated that their tool was piloted as part of a previous study.

#### 3.3.6. Validity and Reliability

Content Validity: Of the 25 tools included, 10 reported some form of content validity, although to varying extents. Dembiński et al. [29] and Paganizza et al. [13] reported development of their tool through systematic review of the literature, and although Paganizza et al. [13] did consult two experts during development, neither study alluded to the use of structured critical analysis of findings. Most reported the support of experts in their field in the development of their tools through peer review, face validity, or expert analysis of the proposed tool questions, suggesting an informal review of the content. Vernero et al. [48] undertook formalised content validation through expert development of the questions and review by both the lay public and volunteers with CD and a special interest in the GFD.

Construct Validity: Only Vernero et al. [48] described undertaking a full formal validation of their tools, with Garg and Gupta [30] mentioning using a previously validated survey. Vernero et al. [48] undertook validation using discriminant validity through the assessment of response variances between two groups: those with well-controlled CD and healthy controls.

Reliability: Roma et al. [42] and Zhou et al. [50] both described formal assessment of tool reliability. The former used the Kappa statistic and test and retest percentage agreement and the latter used Cronbach’s alpha to assess tool reliability.

#### 3.3.7. Generalisability

Generalisability varied between the various tools. Seven tools included local foodstuffs and brands that were specific to the region where the tool was developed, such as barley squash, faggots, Prague ham, Jelly Babies, and Vegemite, and two included local legislation such as food-labelling requirements (Table 2).

### 3.4. Results of Synthesis

Through the systematic review process, a broad range of questions was identified for the assessment of CD knowledge. This may have been due in part to the wide range of target groups included, with knowledge assessment not limited to those with a diagnosis of CD. However, clear themes were identified in the questions asked. Most of the tools identified focused on knowledge surrounding management of a GFD, with only four studies not including an item about the identification of gluten in the diet [29,35,38,45]. All of these studies looked at knowledge of healthcare professionals (HCPs). The study by Dembiński et al. [29] was focused on knowledge among healthcare professionals of the nutritional deficiencies that people with CD may face when following a strict GFD. A general question, “My knowledge of a gluten-free diet is sufficient”, was included. This assumes that the HCPs taking part in the survey were aware of the avoidances required when following a strict GFD.

Only Dembiński [29] and Geiger [29,32], whose target audiences were HCPs, included questions regarding the nutritional concerns for those following a GFD. It is interesting that these questions were not included in more tools as they could potentially be an important knowledge requirement in effectively managing CD.

All CD knowledge assessment questions were extracted from the 17 tools included in the review. Matrix questions identifying gluten in common food products were identified as a single question. The questions were then pooled, grouped into the four main areas mentioned above, and the following nine knowledge domains emerged:

1. General CD knowledge
2. Management of CD
3. Identifying gluten in the diet and as ingredients, e.g., label reading
4. Food labelling and legislation
5. Nutrients and a GFD
6. Food handling practices and training
7. Eating out
8. Medicines, health, and beauty
9. Diagnosing CD

The frequency of questions in each domain from the included tools was summarised (Table 3). The most common questions included related to identifying gluten in the diet, either by selecting the grains that contain gluten or through label reading. The majority of the tools included asked questions regarding knowledge of CD in general, with only two tools asking about knowledge of the nutrients at risk when following a GFD/for those with CD [29,32].

### 3.5. Certainty of Evidence

The studies and assessment tools included in this current review showed high levels of heterogeneity, but as the review is non-data-driven, no formal assessment could be carried out. Additionally, the focus of the review is on the survey tools included in the papers, not the study integrity itself, which leads to the certainty assessment being made according to metrics chosen to evaluate the survey tools. The objective assessment of survey tool composition, content, and format showed that most tools were lacking various important elements being considered during the development process. This limits any statement of certainty as there was no standardisation of tool development between identified studies.

## 4. Discussion

Through the systematic review process, 25 tools were identified to assess knowledge of CD and its management. A predetermined, structured process was used to identify the papers and assess their merit as effective tools. A range of different study types was included in the review. The studies were based in 16 different countries over a period of 21 years and assessed knowledge of CD in a variety of different populations, including health professionals, those with CD, food service workers, and the lay public. The main topics covered in the assessment tools included were general knowledge of CD and its management, including label reading, legislation, eating out, nutrients of concern, and potential non-food sources of gluten, as well as understanding the diagnostic processes for CD.

The purpose of this review was to find a knowledge assessment tool that could be used by healthcare professionals in the clinical and research setting to identify patient knowledge gaps about CD and the GFD that may affect their outcomes. For the tool to effectively satisfy this purpose, it needed to be well designed, tested, and generalisable to the intended population in order to reliably interpret findings when the tool is subsequently used [26]. Consideration of the feasibility of assessment tools, as related to health literacy, readability, and respondent burden, is of paramount importance for both adults and children with CD. However, no formal feasibility testing was carried out in any study included in the review. Health literacy is considered to be the ability to understand, interpret, and act on health information [51,52], and reports have shown health literacy levels to be suboptimal both at school-age and continuing on in to adulthood [53,54]. Hence, the language used in tools designed for the general public should reflect this in order to optimise understanding and participation [55], especially where children are included in the target cohort [56].

The language used in assessment tools should be of a readability level suitable for the target population and of a length that answers the research question sufficiently while not placing undue burden on the user [20,57]. While assessment tools for clinical conditions such as CD may involve some complex clinical terms and jargon, overall, a tool should not have readability assessments higher than a US grade 7-level comprehension [18]. Davis et al. [58] showed that level of education completed can in fact be an inaccurate estimate of reading level. They found the self-reported education level of parents surveyed in an outpatient department being up to a grade-11 level. However, when assessed, reading levels were found to be on average four grade levels lower than this, at around a US 7th- to 8th-grade level. Hence, they found that many health information resources being provided poorly correlated with actual reading level, with only 3% of resources reviewed being written below a 7th-grade level [58]. Previous work has tried to alleviate the respondent burden of complex terms in paediatric health forms by the use of pictures, but this was not a simple fix as children still considered them as needing explanations [59]. The known positive association between higher knowledge levels and greater adherence to GFD among both adults and children highlights how important the use of an appropriate assessment tool is to identify gaps or misconceptions in understanding [15,27,60]. In particular, adherence to a GFD in childhood has also been associated with better growth and quality of life, so inclusion of children in the target population of such assessment tools is crucial [61].

Following the development of assessment tools, undertaking a pilot study allows for testing viability prior to embarking on a full validation study [62]. This enables minor issues with the tool to be resolved prior to commencement and increases the likelihood of success [62]. Carrying out full validity testing then determines whether the measurement tool used actually measures the proposed research concept and can quantify the variables with stable or consistent responses [63]. Although a number of the studies in this current review reported informal pilot testing, no results were reported, and few carried out full validity testing. This thereby limits researcher or clinician confidence in using these published tools. In addition, many of the tools in the review had issues with generalisability, thereby further limiting their applicability.

For an intervention to be of use outside the setting where it was originally developed/evaluated, it must be generalisable to other regions, populations, and clinical settings [64]. In this review, generalisability was limited by the inclusion of questions regarding local food labelling and legislation, support groups, and the inclusion of regional foodstuffs, all of which may vary significantly in different countries. The cut-offs for allowable gluten traces in foods labelled gluten-free, for example, are different in different parts of the world [65], as well as requirements for identifying gluten and potential contamination in packaged goods [10]. A local coeliac society was mentioned in one of the tools. Membership in a society has been associated with better adherence to a GFD, but this resource is not available in all countries and access and format may vary [66,67]. Foodstuffs mentioned in some tools may not be available internationally and may, therefore, be unfamiliar to some target audiences. Consideration in the context of maintaining a GFD must be given to possible variations in recipes of commercial foodstuffs, as well as the possibility of gluten cross-contamination in different countries [68].

No papers included in the review reported all of the pre-determined, necessary feasibility, validity/reliability, and generalisability metrics that would make it a robust and fit-for-purpose tool for the intended populations with CD in the clinical and research setting. The tool that most closely met the requirements for the study was from Vernero et al. [48], which presented a robust tool whose development included piloting and validation. The tool itself covered a broad range of knowledge question areas, covering six of the nine question domains that were developed in the process of data synthesis. Unfortunately, no readability assessments were described, and the tool was not generalisable to other populations due to the use of questions regarding EU-specific legislation and the inclusion of a number of local foodstuffs.

### 4.1. Limitations

Eighteen of the papers that met the basic inclusion criteria were not included as the assessment tool could not be obtained despite repeated attempts to contact authors. Inclusion of these tools would have doubled the number that could be reviewed for inclusion, and their exclusion presents a risk of bias. The lack of standardisation in the format of identified surveys forms a large part of the discussion in this current work; however, it is in itself a study limitation. Inferences as to the best format and content for a coeliac disease knowledge assessment tool are restricted due to their heterogeneity, as well as by a lack of validated assessment tools with which to assess them. Evidence-based metrics were chosen to assess survey tools, but the use of a validated, comprehensive assessment strategy would have enabled more objective summaries and inferences, as well as future comparisons.

### 4.2. Strengths

A robust, transparent review process was undertaken to identify tools used to assess knowledge of CD and its management internationally. Substantial efforts were made to contact the authors of papers where the tool itself was not published in the paper. The tools identified and included in the review were from a variety of centres and with varying target populations. Content synthesis of the included assessment tools allowed for generation of clear themes that may be utilised in future assessment tool development.

## 5. Conclusions

The maintenance of a strict GFD is key to effective management of CD, with better knowledge supporting autonomy in self-management and adherence [69]. However, there are wide variations in practice for the education provided to people with CD and their wider communities [70]. Hence, assessing knowledge of CD and its management and identifying any gaps would be essential for planning and development of future education resources. The results of this current review will inform further research to address the knowledge gap of having no suitable CD knowledge assessment tool available in the literature.

## Figures and Tables

**Table 1:** Summary of the characteristics of studies and their participants.

First Author	Data Collected	Country	Recruitment Setting	Study Design	Target Cohort	Participants
Alorayyidh [27]	2019–2020	Saudi Arabia	One hospital	Cross-sectional	Adults with CD	90
Ciacci [28]	2021	Italy	-	Cross-sectional	Young Adults with CD	20
Dembiński [29]	2021	Poland	Advert: medical societies	Cross-sectional	Drs., dietitians, nurses	Medical students: 283 Doctors: 101 Nurses: 29 Dietitians: 17
Garg [30]	2011–2012	India	One tertiary care hospital OPD	Cross-sectional	Parents of children with CD	134
Garipe [31]	2011	Argentina	Food stores in three regions	Cross-sectional	Food shop employees	142
Geiger [32]	2013	US	State specific ^ dietitian academy members	Cross-sectional	Dietitians	405
Gutowski [33]	2012–2015	Canada	Provincial CD cohort	Longitudinal	Adults with CD	144
Haboubi [34]	-	UK	Regional health board employee mailing list	Cross-sectional	Dietitians, doctors, nurses, managers/admin staff	Doctors: 47 Nurses: 37 Dietitians: 25 Managers/admin: 3
Harvey [35]	2021–2022	Germany, Italy, Spain, and the US	Adelphi Disease Specific Programme™ for CeD	Cross-sectional	Gastroenterologists (GIs) and primary care physicians (PCPs)	178 GIs 100 PCPs
Howard [36]	2020	UK	Two food banks	Cross-sectional	Adult Food bank volunteers	20
Khafagy [37]	-	Saudi Arabia	Restaurants in two cities	Cross-sectional	Restaurant owners or employees	126
Kozhakhmetova [38]	-	Kazakhstan	Nationally via email using National Center of Public Health database	Cross-sectional	Physicians working in public and private medical organisations	232
Mehtab [39]	2021–2022	India	Single coeliac clinic	Cross-sectional	Adults with CD	978
Meyer [40]	-	Germany	National Coeliac Society	RCT	Adults with CD	64
Paganizza [13]	2016	Italy	One tertiary care hospital OPD	Cross-sectional	Adults with CD	104
Riznik [41]	2017–2019	Central Europe *	-	Cross-sectional	Children/parents and adults with CD, HCP **	HCP: 1381 Children CD: 63 Parents: 892 Adults CD: 1307
Roma [42]	2008	Greece	One children’s hospital OPD	Cross-sectional	Children with CD and parents	73
Silvester [43]	2011–2012	Canada	Advert: support group, GFD retail outlets, OPD	Cross-sectional	Adults with CD, adults non-CD on GFD	CD: 222 Non-CD: 38
Simpson [44]	2010	US	CD support groups, OPD, areas in one city, mailing lists	Cross-sectional	Adult public, chefs, adults with CD	Public: 861 Chefs: 430 CD: 790
Tan [45]	-	Netherlands	Gastroenterologists and Dentists	Cross-sectional	Gastroenterologists and Dentists	107 Gastroenterologists, 103 Dentists
Uršulin-Trstenjak [46]	2017	Croatia	Regional community social media post	Cross-sectional	Adult public	210
Vázquez-Polo [47] #	2024	Spain		Longitudinal	Children public	~144
Vernero [48]	2020–2021	Italy	20 regional hospital OPD’s	Experimental	Adults with CD, adult public	CD: 52 Public: 54
Weisbrod [49]	-	US	One tertiary care hospital OPD	Cross-sectional	Parents of children with CD	37
Zhou [50]	-	Canada	Two tertiary care, university-affiliated hospitals	Cross-sectional	Food handlers	72

US = United States, OPD = out-patient departments, GFD = gluten-free diet, CD = coeliac disease, RCT = randomised controlled trial, HCP = healthcare professionals, - = not reported. * Croatia, Hungary, Germany, Italy, and Slovenia. ** primary care physicians, paediatricians, gastroenterologists, dietitians, paediatric residents, neurologists, ophthalmologists, anaesthesiologists, dermatologists, dentists, infectious disease specialists, psychiatrists. ^ Alaska, Colorado, Connecticut, Delaware, Montana, Nebraska, and North Dakota. # Protocol not yet published.

**Table 2:** Knowledge assessment tool characteristics, design and testing.

First Author	Number of Questions and Food Items	Format of Questions	Readability	Piloted	Content Validity	Validation	Reliability	Generalisability Issues
Alorayyidh [27]	39 (4 sections) Knowledge: 7 Knowledge (GFD-KS): 17 * Excluded: 15	Yes/No MCQ	NS	Yes (n = 50)	Developed by 3 dietitians	NS	NS	
Ciacci [28] ^	49 Knowledge: 13 Excluded: 36	True/False MCQ	NS	NS	NS	NS	NS	
Dembiński [29]	14 Knowledge: 12 Excluded: 2	Yes/No MCQ	NS	NS	Developed using previous systematic review	NS	NS	
Garg [30]	5 GCF: 1 (5 items) Knowledge: 4	Free text	NS	Used previously pretested survey	NS	Used previously validated (n = 20) and pretested survey	NS	
Garipe [31]	7 Knowledge: 6 Excluded: 1	Yes/No	NS	Yes	NS	NS	NS	
Geiger [32]	12 Knowledge: 12	MCQ	Informal, results not reported	Yes	NS	NS	NS	Local restaurants, shops, and support groups discussed
Gutowski [33]	3 GCF: 3 (75 items)	Yes/No	NS	NS	Developed by experts	NS	NS	Food terms/names
Haboubi [34]	4 GCF: 2 (64 items) Contamination: 1 (12 items) Knowledge: 1 (9 items)	Yes/No	NS	NS	NS	NS	NS	Food terms/names
Harvey [35] ^	Knowledge: 2 Excluded: 6	Yes/No MCQ Open	NS	NS	NS	NS	NS	
Howard [36]	5 GCF: 2 (25 items) Excluded: 3	MCQ	Informal, results not reported	Yes	Peer-reviewed	Stated as not performed	NS	Food terms/names
Khafagy [37]	20 Knowledge: 3 Excluded: 17	MCQ	NS	NS	NS	NS	NS	
Kozhakhmetova [38]	15 Knowledge: 9 Excluded: 6	Yes/No MCQ	NS	NS	NS	NS	NS	
Mehtab [39]	72 Knowledge: 4 Excluded: 68	Yes/No	NS	Yes (n = 15)	Developed by an expert panel	NS	NS	
Meyer [40]	22 GCF: 1 (46 items) Knowledge: 21	Yes/no MCQ	NS	NS	NS	Stated as not performed	Stated as not performed	Food terms/names
Paganizza [13]	31 GCF: 17 Knowledge: 14	True/false MCQ	NS	NS	Developed from content synthesis of literature and expert review	NS	NS	Food terms/names, parts-per-million legislation
Riznik [41]	HCP—36 GCF: 1 Knowledge: 26 Excluded: 10 Children CD/parents—36 Knowledge: 14 Excluded: 22	Yes/no MCQ	NS	Yes	Peer reviewed	NS	NS	
Roma [42]	15 knowledge: 15	Yes/No	Informal, results not reported	NS	NS	NS	Yes	Food terms/names Local society
Silvester [43]	6 GCF: 1 (10 items) Knowledge: 5	True/false MCQ Free text	Informal, results not reported	Yes	Developed by expert panel	NS	NS	
Simpson [44]	31 GCF: 1 (7 items) Knowledge: 2 Excluded: 28	Yes/No MCQ	NS	NS	NS	NS	NS	Rates of regional coeliac disease included
Tan [45]	4 sections (2-knowledge 19) Knowledge: 4 Excluded: 15	True/False/don’t know	NS	Yes (n = 3 GI, n = 4 Dentist)	NS, ‘self-developed’	NS	NS	
Uršulin-Trstenjak [46]	22 Knowledge: 18 Excluded: 4	Yes/No MCQ	NS	NS	NS	NS	NS	
Vázquez-Polo [47] #	32 (8 competencies) Knowledge: 14 Excluded: 18	Yes/No MCQ Open	NS	NS	NS	NS	NS	
Vernero [48]	10 Knowledge: 10	MCQ	NS	Yes	Yes	Yes	No	EU specific legislation and items, food terms/names
Weisbrod [49]	7 GCF: 1 (12 items) Knowledge: 6	True/false MCQ	NS	NS	NS	NS	NS	Country specific legislation, information sources, food labelling
Zhou [50]	45 Knowledge: 40 Excluded: 5	True/false MCQ	Yes	Yes (n = 5)	Developed by gastroenterologists and dietitians with expertise in GFD	NS	Yes	

GCF = gluten-containing food lists, MCQ = multiple choice question, NS = not stated. * Already included in Silvester [43]. ^ presented as a poster presentation/abstract, no additional information provided. # Protocol not yet published.

**Table 3:** Frequency of item topics in each knowledge assessment tool.

First Author	General CD Knowledge	Management	Identifying Gluten in Diet + Ingredients	Food Labelling + Legislation	Nutrients in Gluten -Free Diet	Food-Handling Practices and Training	Eating Out	Medicines, Health, and Beauty	Diagnostics for CD	Knowledge Results
Alorayyidh [27]	√	√								5/7 (71%)
Ciacci [28]	√	√	√	√		√			√	No results included
Dembiński [29]	√				√					Items: 25–73% correct
Garg [30]	√	√	√							No results included
Garipe [31]	√		√			√				Full score: 3.5%
Geiger [32]	√	√	√		√		√	√	√	Items: 85–98% correct
Gutowski [33]			√							Median: 23/25 (92%)
Haboubi [34]			√			√				Doctors: 70% Nurses: 68% Dietitians: 85% Managers/admin: 55%
Harvey [35]		√							√	Gastroenterologists 57.5–81.7% correct Primary care physicians 35.4- 78.3% correct
Howard [36]	√		√			√				Mean 10.5/20 (53%)
Khafagy [37]	√		√							34.1% correctly identified gluten
Kozhakhmetova [38]	√	√							√	14.7 ± 6.9/38
Mehtab [39]	√	√	√	√						
Meyer [40]			√				√	√	√	10.5/14 (75%) 23.5/26 (90%)
Paganizza [13]	√	√	√	√				√		Mean 18/31 (58%)
Riznik [41]	√	√	√						√	HCPs: 51% Children CD: 54% Parents: 58% Adults CD: 55%
Roma [42]	√	√	√				√			>13/15 = 42.5%
Silvester [43]			√							CD: items 7–99% correct Non-CD: 3–97% correct
Simpson [44]	√		√							No results included
Tan [45]	√									Gastroenterologists: 25.2–65.4% correct Dentists: 12.9–72% correct
Uršulin-Trstenjak [46]	√	√	√	√						Items: 30–99% correct
Vázquez-Polo [47]	√	√	√							
Vernero [48]		√	√	√		√	√	√		CD: median 6/10 (60%) Public: median 2/10 (20%)
Weisbrod [49]			√	√			√	√		Full score: 11%
Zhou [50]	√	√	√			√				26.6/35 (75.9% ± 13.4%)
Frequency	18	14	20	6	2	6	5	5	6	

## Data Availability

No new data were created or analyzed in this study. Data sharing is not applicable to this article.

## Appendix A. Summary of the Systematic Review Search Criteria and Strategies

**Medline 1 March 24**

**Medline 25 February 22–1 March 24**

1	Celiac Disease/	22,170
2	c*eliac.ti.	19,017
3	Knowledge/ or Knowledge Management/ or Health Knowledge, Attitudes, Practice/	1,034,655
4	knowledg*.tw.	965,614
5	informat*.tw.	1,670,467
6	assess*.tw.	3,973,535
7	measur*.tw.	4,283,476
8	survey*.tw.	875,303
9	questionnaire*.tw.	697,221
10	1 or 2	27,021
11	3 or 4 or 5	2,552,483
12	6 or 7	7,141,809
13	8 or 9	1,412,318
14	10 and 11 and 12 and 13	107

**Embase 6 March 2024**

1	Celiac Disease/	43,776
2	c*eliac.ti.	26,787
3	knowledge/ or knowledge base/ or knowledge management/	1,251,846
4	knowledg*.tw.	1,225,324
5	informat*.tw.	2,175,309
6	assess*.tw.	5,683,661
7	measur*.tw.	5,711,023
8	survey*.tw.	1,160,249
9	questionnaire*.tw.	1,005,128
10	1 or 2	48,188
11	3 or 4 or 5	3,240,877
12	6 or 7	9,788,779
13	8 or 9	1,942,371
14	10 and 11 and 12 and 13	282

**CINAHL 10 March 2024**

S5	S1 AND S2 AND S3 AND S4	(51)
S4	TI (assess* OR measur*) OR AB (assess* OR measur*)	(1,638,044)
S3	TI (survey* OR questionnaire*) OR AB (survey* OR questionnaire*)	(565,473)
S2	TI (knowledg* OR informat*) OR AB (knowledg* OR informat*)	(654,155)
S1	TI (celiac OR coeliac) OR AB (celiac OR coeliac)	(6725)

**PsychInfo 10 March 2024**

**Searches**	**Results**	**Type**
1	Celiac Disease/	296
2	c*eliac.ti.	317
3	Knowledge/ or Knowledge Management/ or Health Knowledge, Attitudes, Practice/	4828
4	knowledg*.tw.	375,633
5	informat*.tw.	542,814
6	assess*.tw.	934,618
7	measur*.tw.	949,769
8	survey*.tw.	381,287
9	questionnaire*.tw.	335,768
10	1 or 2	384
11	3 or 4 or 5	851,357
12	6 or 7	1,577,637
13	8 or 9	660,244
14	10 and 11 and 12 and 13	9

**SCOPUS 10 March 2024**

(TITLE(c*eliac) AND TITLE-ABS-KEY(knowledg* OR informat*) AND TITLE-ABS-KEY(assess* OR measur*) AND TITLE-ABS-KEY(survey* OR questionnaire*)) = 146

**OpenGrey (via DANS) 28 February 22**

Search: Celiac = 17 results = 0 relevant

Search: Coeliac = 1 result = 0 relevant
